# Vaccination coverage and the factors influencing routine childhood vaccination uptake among communities experiencing disadvantage in Vellore, southern India: a mixed-methods study

**DOI:** 10.1186/s12889-021-11881-8

**Published:** 2021-10-07

**Authors:** Mark Rohit Francis, J. Pekka Nuorti, Kirsi Lumme-Sandt, Rajeev Zachariah Kompithra, Vinohar Balraj, Gagandeep Kang, Venkata Raghava Mohan

**Affiliations:** 1 Health Sciences Unit, Faculty of Social Sciences, Tampere University, Tampere, Finland; 2 Infectious Diseases and Vaccines Unit, Department of Health Protection, National Institute for Health and Welfare, Helsinki, Finland; 3 Well Baby Immunization Clinic, Department of Pediatrics Unit – I, Christian Medical College, Vellore, Tamil Nadu India; 4 Department of Community Health, Christian Medical College, Vellore, Tamil Nadu India; 5 Division of Gastrointestinal Sciences, Christian Medical College, Vellore, Tamil Nadu India

**Keywords:** Mission Indradhanush, Communities experiencing disadvantage, Parental perceptions, Childhood vaccination, Vellore, India

## Abstract

**Background:**

In 2015, the Vellore district in southern India was selected for intensified routine immunization, targeting children from communities experiencing disadvantage such as migrant, tribal, and other hard-to-reach groups. This mixed-methods study was conducted to assess routine immunization coverage and the factors influencing childhood vaccination uptake among these communities in Vellore.

**Methods:**

We conducted a cross-sectional household survey (*n* = 100) and six focus group discussions (*n* = 43) among parents of children aged 12–23 months from the known communities experiencing disadvantage in Vellore during 2017 and 2018. Multivariate logistic regression was conducted to examine associations between the parental characteristics and children’s vaccination status in the household survey data; the qualitative discussions were analyzed by using the (previously published) “5As” taxonomy for the determinants of vaccine uptake.

**Results:**

In the household survey, the proportions of fully vaccinated children were 65% (95% CI: 53–76%) and 77% (95% CI: 58–88%) based on information from vaccination cards or parental recall and vaccination cards alone, respectively. Children whose mothers were wage earners [Adjusted prevalence odds ratio (aPOR): 0.21, 95% CI = 0.07–0.64], or salaried/small business owners [aPOR: 0.18, 95% CI = 0.04–0.73] were less likely to be fully vaccinated than children who had homemakers mothers. In the focus group discussions, parents identified difficulties in accessing routine immunization when travelling for work and showed knowledge gaps regarding the benefits and risks of vaccination, and fears surrounding certain vaccines due to negative news reports and common side-effects following childhood vaccination.

**Conclusions:**

Vaccination coverage among children from the surveyed communities in Vellore was suboptimal. Our findings suggest the need to target children from Narikuravar families and conduct periodic community-based health education campaigns to improve parental awareness about and trust in childhood vaccines among the communities experiencing disadvantage in Vellore.

**Supplementary Information:**

The online version contains supplementary material available at 10.1186/s12889-021-11881-8.

## Background

The Indian Universal Immunization Program (UIP) is the largest public health initiative of its kind, tasked with vaccinating nearly 27 million children every year [1]. The UIP currently provides free vaccines against tuberculosis (BCG), poliomyelitis (OPV and IPV), diphtheria, pertussis, tetanus, *H. influenzae* type b, hepatitis B (pentavalent), measles-rubella (MR), rotavirus diarrhea, Japanese Encephalitis (in endemic districts) and pneumococcal diseases (in some Indian states) [2]. India was certified polio-free in 2014 as a result of a decade-long intensification of polio immunization activities [3]. Following this successful polio eradication campaign, the Indian Government launched the Mission Indradhanush (MI) campaign in 2015 to increase full immunization coverage (children aged 12–23 months who receive one dose each of BCG and measles vaccines and three doses of pentavalent and OPV) in the poorest performing districts to 90% by 2020 [4]. While administrative reports suggested improved full immunization coverage after the first two phases of MI, the recently concluded National Family Health Survey (NFHS-4, 2015–16) reports increased immunization coverage for all Indian states except Haryana, Himachal Pradesh, Uttarakhand, Maharashtra, and Tamil Nadu which warrants further investigation [5, 6].

Tamil Nadu has traditionally had high full immunization coverage and is the only Indian state which provides a financial incentive to economically disadvantaged mothers whose children have received three pentavalent doses [7]. The NFHS-4 estimates full immunization coverage at 70% for Tamil Nadu during 2015–16, compared with 81% during 2005–06 (NFHS-3) [8]. Eight out of thirty-eight districts in Tamil Nadu were selected for intensified routine immunization (including the organization of special immunization sessions, enhanced community engagement and mobilization, and increased accountability at all levels of program implementation) through the MI campaign in 2015 [9]. Vellore was one of the eight “MI districts” in Tamil Nadu, and NFHS-4 reported full immunization coverage of 74% for Vellore with an important urban-rural difference in coverage (78% versus 69%, respectively) [9, 10]. However, a recent survey among young children in rural Vellore reports a full immunization coverage of 84%, close to the prescribed MI target of 90% [11]. An important MI objective involved targeting children from disadvantaged communities such as migrant, tribal, and other hard-to-reach groups [9]. While there is no similar data from Vellore for comparison, a recent review of studies from other parts of India suggests that full immunization coverage among children in communities experiencing disadvantage varies widely from 31 to 89% [12].

Timely and region-specific estimates of routine immunization coverage among children from disadvantaged communities can identify potential inequities in service delivery or uptake and inform targeted interventions to tackle the barriers to vaccination uptake in such settings. With this mixed-methods study, we aimed to assess routine immunization coverage and the factors influencing childhood vaccination uptake among communities experiencing disadvantage in Vellore.

## Methods

### Study setting and communities

This mixed-methods study was conducted in the Vellore district of Tamil Nadu, southern India. The district has a population of approximately 4 million, of which 43% of residents live in urban, and 57% live in rural areas (2011 Census). There are nearly half a million children under six years of age and literacy is approximately 80% (2011 Census). Routine immunization is generally provided to children in primary health centers, childcare centers (Anganwadis), or the government district hospital at no cost to parents. There are private clinics and hospitals in Vellore that provide vaccinations for a fee, generally using the Indian Academy of Pediatrician (IAP) immunization schedule [13].

Parents or primary caretakers (adults involved in caring for children and knowledgeable of their immunization history) of children under two years of age from the known communities experiencing disadvantage in Vellore such as Narikuravar, Irular, stone quarry, and brick kiln worker communities participated in our study. The Narikuravar are a semi-nomadic tribe of Tamil Nadu similar in origin to the Romani or Roma communities of Europe [14]. This community has low literacy, poor access to public welfare services such as health care, and limited sources of income [15]. The Irular are a large tribal group of Tamil Nadu characterized by low literacy, extreme poverty, and facing a degree of cultural and geographic isolation [16, 17]. Brick kiln and stone quarry workers are generally migrants from adjoining districts in Tamil Nadu or the neighboring Indian States and reside in suboptimal conditions, have poorer health than the general population, and limited or no access to public welfare services [18, 19].

### Cross-sectional survey

The methods of the cross-sectional survey are organized in accordance with the STROBE guidelines for observational studies (Supplemental Table 1).

#### Study participants

Parents of children aged 12–23 months (henceforth called eligible children) were eligible to participate in the cross-sectional household survey conducted to assess routine immunization coverage and the factors associated with childhood vaccination uptake during December 2017 and January 2018. The age range of eligible children (12–23 months) was set to assess the coverage of vaccines during their first year of life, following expanded programme on immunization (EPI) and UIP guidelines [9, 20]. The vaccination status of children aged 12–23 months is an important indicator widely used to evaluate national and regional immunization programs in India and other developing countries [1, 20–22].

#### Sample size

Since estimates of the proportions of fully vaccinated children (children who received one dose of BCG and measles and three doses each of pentavalent and OPV vaccines) were not available for the different disadvantaged communities in southern India, we used an anticipated proportion of 50% fully vaccinated children with absolute precision of ±10% and inflating for 15% non-response, to estimate that 110 children needed to be surveyed.

#### Recruitment

Due to the lack of a pre-existing sampling frame, we surveyed all households with eligible children using an adaptation of traditional snowball sampling, where contacted respondents typically refer one or more respondents to the study [23]. This approach was used to identify additional communities once initial contact with at least one of each community type was made since Narikuravar and Irular settlements are especially difficult to locate and recruit for research [15–17]. Twelve Narikuravar, 16 Irular, 3 brick kiln, and 3 stone quarry settlements in Vellore were covered in the cross-sectional survey.

#### Data collection

Data for the cross-sectional survey were collected by trained field workers using a pre-tested, interviewer-administered questionnaire after obtaining written informed consent from the parents of eligible children. The questionnaire consisted of three major sections that captured information on child and parents’ socio-demographic characteristics, children’s immunization history and the reasons for non-vaccination, and parental awareness, attitudes, and concerns regarding routine vaccination (See supplemental material for the survey questionnaire). We collected information on socio-demographic characteristics such as parents’ age, education, occupation, household type, caste and religion, and child characteristics such as age, gender, and place of birth. The section collecting children’s immunization history was adapted from the EPI cluster survey questionnaire [20]; Information on childhood vaccinations was recorded from vaccination cards, if available, or through parental recall when vaccination records were unavailable. We outlined the section on parents’ awareness, attitudes, and concerns regarding routine vaccines using the “5As” taxonomy for the determinants of vaccine uptake - a published framework developed to provide a practical nomenclature to organize the possible root causes of vaccination coverage gaps in diverse settings [24]. This section included questions on “Access” – mode of travel to vaccination centers (a proxy for distance to vaccination centers), “Affordability” – the timing of immunization services (an opportunity cost, since routine vaccines, are provided free of cost), “Awareness” – familiarity with the UIP immunization schedule, “Acceptance” – reported hesitancy about childhood vaccines, and “Activation” – receipt of the information on the UIP schedule during antenatal visits, and a monetary incentive for completing the pentavalent vaccination series. The definitions of the 5As are presented in Table 1. The questionnaire was translated to local vernacular (Tamil) and programmed using the KoBo Toolbox suite, an open-source application for mobile data collection [25].

**Table 1:** Definitions of the “5As” taxonomy for the determinants of vaccine uptake domains [21]

“5As” domains	Definition
**Access**	The ability of individuals to be reached by, or to reach, recommended vaccines
**Affordability**	The ability of individuals to afford vaccination, both in terms of financial and non-financial costs (e.g. time)
**Awareness**	The degree to which individuals have knowledge of the need for, and availability of, recommended vaccines and their objective benefits and risks
**Acceptance**	The degree to which individuals accept, question or refuse vaccination
**Activation**	The degree to which individuals are nudged towards vaccination uptake

#### Statistical analysis

Data from the cross-sectional survey were entered real-time on the “KoBoCollect” application for Android™ devices [26]. Range checks, skip patterns, and pictures of children’s vaccination cards were programmed into the interface to minimize data-entry errors. These data were reviewed for completeness, and birth dates and vaccinations were verified using photos of children’s vaccination cards. Descriptive analyses were conducted to summarize the distribution of the study variables using frequencies, percentages, means, and standard deviations where appropriate. Next, the proportions of children aged 12–23 months vaccinated with the recommended UIP doses were estimated using information from 1) vaccination cards or parental recall and 2) vaccination cards alone, following EPI recommendations. Although estimates from documented sources such as vaccination cards or health-facility records are preferable, combining vaccination cards and parental recall information provides a “crude” estimate of vaccination coverage, which is useful in settings where immunization cards are not commonly available [20]. We also calculated the sensitivity and specificity of parental recall and vaccination card information to classify children’s vaccination status for the subset of children with a vaccination card available during the survey. Univariate associations between the independent variables, including socio-demographic and non-socio-demographic characteristics (parents’ awareness, attitudes, and concerns regarding routine vaccines) and children’s vaccination status (based on parental recall or vaccination cards), were assessed using Chi-square or Fisher’s exact tests. Children’s vaccination status was categorized as “fully vaccinated” or “under-vaccinated,” based on EPI and UIP recommendations; A fully vaccinated child was one who had received one dose of BCG and measles-containing vaccine (monovalent measles or Measles-Rubella) and three doses each of pentavalent and OPV vaccines by 12 months of age [2, 20].

The independent variables associated with children’s vaccination status at the *p* < 0.05 level in the univariate analyses were included in a multivariate logistic regression model. Multicollinearity between independent variables in the multivariate model was assessed by estimating the variance inflation factor (VIF) [27]. Since none of the VIF values reached 10 and the mean VIF of the multivariate model was 3.28, there was no evidence of multicollinearity between variables [27]. Hosmer and Lemeshow’s goodness-of-fit test was used to evaluate the fit of the multivariate regression model [28]. The multivariate analysis findings are presented as adjusted Prevalence Odds Ratios (aPORs) with 95% CIs derived from design-adjusted standard errors. We considered multivariate associations with a *p* < 0.05 as statistically significant. All analyses accounted for the clustering of children in the individual settlements (34 in total), using the “*svy*” package in STATA (version 14, StataCorp LP, College Station, TX, USA).

### Focus group discussions

#### Study participants

Parents of children aged 12–23 months who participated in the cross-sectional survey were eligible to join the focus group discussions conducted to investigate the barriers and facilitators of childhood vaccination uptake among these populations.

#### Sample size

The focus group discussions were conducted to assess community norms and parental perceptions surrounding vaccinations for their children. Due to logistical constraints, we determined a priori that two FGDs with 8–10 parents per meeting would be conducted in the Narikuravar, Irular, and stone quarry communities for this qualitative investigation (6 FGDs in total).

#### Recruitment

Purposive sampling was used to recruit parents for the focus group discussions conducted in December 2017. Only parents who participated in the cross-sectional survey and indicated their willingness to join follow-up discussions were contacted by the trained field workers.

#### Data collection

A pre-tested thematic guide containing open-ended questions exploring aspects such as perceptions on childhood vaccination and immunization safety, parent–healthcare worker interactions, and suggestions for improving routine immunization services was used for the FGDs. The thematic guide was developed in English, translated to local vernacular (Tamil), and modified using feedback from a pilot FGD conducted in a community that was not part of the survey (see supplemental material for the FGD guide). The FGDs were conducted in Tamil by a field supervisor with extensive experience in community engagement and fieldwork in the study region. Separate FGDs were held with mothers and fathers to ensure their free participation. The FGDs were audio-recorded after obtaining verbal consent from the participating parents. The lead investigator (MRF) was present as a facilitator during all the meetings and recorded written observations relevant to the qualitative analysis. Important responses to the different FGD topics were clarified during each meeting by the field supervisor and lead investigator to better understand them within the sociocultural contexts of the participating communities.

#### Data analysis

Anonymized audio transcripts from the FGDs were translated into English, and the responses were entered in Microsoft Word for initial analysis. Data were reduced using open coding, and common categories (sub-themes) were identified for each question inductively by the first author (MRF). The sub-themes and associated responses were then mapped to the “5As” taxonomy domains to triangulate the findings of the focus group discussions with those from the household survey [24]. The mapping of sub-themes to the “5As” domains is presented in Table 2. A co-author (KLS) checked the consistency and relevance of the mapped sub-themes and responses. Quotes from participants have been used to support the findings where appropriate and additional text for clarification placed within square brackets, as necessary. The mapping and organization of sub-themes and associated responses to the “5As” domains was performed using Microsoft Excel.

**Table 2:** Mapping of sub-themes from the focus group discussions to the “5As” taxonomy domains [21]

“5As” domains	Sub-themes (from open-coding)
**Access**	Good access to vaccines, travel out of town as reason for missed or delayed doses, time of travel out of town
**Affordability**	Convenient timing of immunization sessions, free vaccination provided by the government a benefit
**Awareness**	Benefits of vaccination, names of vaccines (or diseases prevented), knowledge sharing by health care workers, other sources of vaccination information, limited awareness of benefits/risks of vaccination, more information requested
**Acceptance**	Positive view of vaccines in general, vaccination as a social responsibility, influence of health care worker on parents, family or peer influence on attitudes, impact of negative news on parental attitudes, experiences with vaccination, fear of vaccine side-effects
**Activation**	Government ads and campaigns, prompts and reminders by health care workers, provisions for delayed doses, financial incentives for vaccination
**Uncategorized**	Choice of vaccination centers

## Results

### Cross-sectional survey

A total of 100 children aged 12–23 months were included in the household surveys (two families declined to participate, response proportion = 98%). The mean (SD) age of children was 18.7 (3.4) months; 53% of children belonged to Narikuravar communities and 47% to Irular, stone quarry, and brick kiln communities (Table 3). Most participants (89%) were mothers, 46% of all mothers had no formal education, and 51% were homemakers. Almost all parents (95%) agreed that immunization was important to keep their children healthy, and a little more than half (56%) reported that they were familiar with the recommended immunization schedule for their children.

**Table 3:** Characteristics of the study participants in the household survey and their association with the vaccination status of children among communities experiencing disadvantage in Vellore, southern India (*N* = 100)

Characteristic	Categories	***N*** (%)	Fully vaccinated, ***n*** (%)	Under vaccinated, ***n*** (%)	***p-***value*
***Socio-demographic***					
**Child’s gender**	Male	53 (53.0)	35 (66.0)	18 (34.0)	0.805
	Female	47 (47.0)	30 (63.8)	17 (36.2)	
**Place of birth**	Public facility	78 (78.0)	52 (66.7)	26 (33.3)	0.198^†^
	Private facility	12 (12.0)	9 (75.0)	3 (25.0)	
	Home	10 (10.0)	4 (40.0)	6 (60.0)	
**Mother’s education**	No formal education	46 (46.0)	23 (50.0)	23 (50.0)	**0.029**
	Primary school or higher	54 (54.0)	42 (77.8)	12 (22.2)	
**Father’s education**	No formal education	39 (39.0)	20 (51.3)	19 (48.7)	0.097
	Primary school or higher	61 (61.0)	45 (73.8)	16 (26.2)	
**Mother’s occupation**	Homemaker	51 (51.0)	43 (84.3)	8 (15.7)	**< 0.001**
	Wage earner	17 (17.0)	10 (58.8)	7 (41.2)	
	Salary earner/small business owners	32 (32.0)	12 (37.5)	20 (62.5)	
**Father’s occupation**	Unemployed/wage earner^a^	62 (60.0)	47 (76.7)	15 (23.3)	**0.005**
	Salary earner/small business owners	38 (38.0)	18 (47.4)	20 (52.6)	
**Religion**	Hindu	83 (83.0)	57 (68.7)	26 (31.3)	0.130
	Others	17 (17.0)	8 (47.1)	9 (52.9)	
**Community type**	Narikuravar	53 (53.0)	27 (50.9)	26 (49.1)	**0.022**
	Other communities^b^	47 (47.0)	38 (80.9)	9 (19.1)	
**Type of dwelling**	Mud/semi-cemented	45 (45.0)	28 (62.2)	17 (37.8)	0.503
	Cemented	55 (55.0)	37 (67.3)	18 (32.7)	
**Vaccination card**	Not available	49 (49.0)	26 (53.1)	23 (46.9)	**0.006**
	Yes	51 (51.0)	39 (76.5)	12 (23.5)	
***Non-socio-demographic***					
**Mode of travel to immunization facility (proxy for distance to facility)**	Walking	54 (54.0)	32 (59.3)	22 (40.7)	0.200
	Private or public transport	46 (46.0)	33 (71.7)	13 (28.3)	
**I think immunization is important to keep my child healthy**	Not agree (N)	5 (5.0)	1 (20.0)	4 (80.0)	0.075^†^
	Agree (SA,A)^c^	95 (95.0)	64 (67.4)	31 (32.6)	
**I am familiar with the recommended immunization schedule for children**	Not agree (N,DA, SDA)	44 (44.0)	22 (50.0)	22 (50.0)	**0.035**
	Agree (SA,A)	56 (56.0)	43 (76.8)	13 (23.2)	
**Reported hesitancy about one or more childhood vaccines**	Hesitant (N,SH,VH)	22 (22.0)	14 (63.6)	8 (36.4)	0.869
	Not hesitant (NH,NTH)^d^	78 (78.0)	51 (65.4)	27 (34.6)	
**Received information about the recommended immunization schedule during antenatal visits**	No or not sure	9 (9.0)	2 (22.2)	7 (77.8)	**0.007**
	Yes	91 (91.0)	63 (69.2)	28 (30.8)	
**Incentive for receiving three doses of pentavalent vaccine**	No or not sure	51 (51.0)	27 (52.9)	24 (47.1)	**0.015**
	Yes	49 (49.0)	38 (77.6)	11 (22.4)	

* The *p*-values account for clustering among surveyed children; Boldface indicates *p* < 0.05

^†^ *P*-value from Fisher’s exact test due to the low cell counts

^a^ *n* = 2 fathers were unemployed during the survey

^b^ Other communities include the Irular, brick kiln, and stone quarry worker communities

^c^ *SA* Strongly agree, *A* Agree, *N* Neutral, *DA* Disagree, *SDA* Strongly disagree

^d^ *SH* Strongly hesitant, *VH* Very hesitant, *N* Neutral, *NH* Not hesitant, *NTH* Not too hesitant

Of the children included, 51% had a vaccination card, and the rest reportedly had a vaccination card that could not be produced during the survey. Vaccination coverage using information from vaccination cards or parental recall (*n* = 100) was 97% (95% CI: 92–99%) for BCG, and 81% (95% CI: 70–89%) and 75% (95% CI: 65–83%) for the third dose of pentavalent and measles vaccination respectively (Table 4). Among children with a vaccination card (*n* = 51), coverage of BCG, third dose of pentavalent and measles vaccination was 94% (95% CI: 85–98%), 90% (95% CI:76–96%), 90% (95% CI:77–96%) respectively. The proportions of fully vaccinated children were 65% (95% CI: 53–76%) and 77% (95% CI: 58–88%) for information based on either vaccination cards or parental recall and vaccination cards alone, respectively (Table 4). The sensitivity and specificity of parental recall (to classify their child’s vaccination status) using vaccination card information as the gold standard for children with a card (*n* = 51) was 100 and 58%, respectively.

**Table 4:** Coverage and vaccination status of children aged 12–23 months among communities experiencing disadvantage in Vellore, southern India

Vaccine antigen	Card or parental recall (***n*** = 100)		Card only (***n*** = 51)	
	Number vaccinated	Proportion (95% CI)^b^	Number vaccinated	Proportion (95% CI)
**BCG**	97	97.0 (92.4–98.8)	48	94.1 (84.8–97.9)
**Pentavalent- 1**	90	90.0 (83.0–94.3)	48	94.1 (85.2–97.8)
**Pentavalent- 2**	86	86.0 (78.3–91.3)	50	98.0 (89.7–99.7)
**Pentavalent- 3**	81	81.0 (70.2–88.5)	46	90.2 (76.0–96.4)
**OPV- 1**	92	92.0 (84.6–96.0)	50	98.0 (89.7–99.7)
**OPV- 2**	86	86.0 (78.3–91.3)	50	98.0 (89.7–99.7)
**OPV- 3**	80	80.0 (68.9–87.8)	45	88.2 (74.8–95.0)
**Measles or MR**	75	75.0 (65.3–82.7)	46	90.2 (76.9–96.2)
**Fully vaccinated**^a^	65	65.0 (52.5–75.8)	39	76.5 (58.2–88.4)

^a^ Children who received one dose of BCG, three doses each of OPV and pentavalent and one dose of measles or MR by 12 months of age

^b^ 95% Confidence Intervals (CIs) account for clustering among surveyed children

**Fig. 1:**
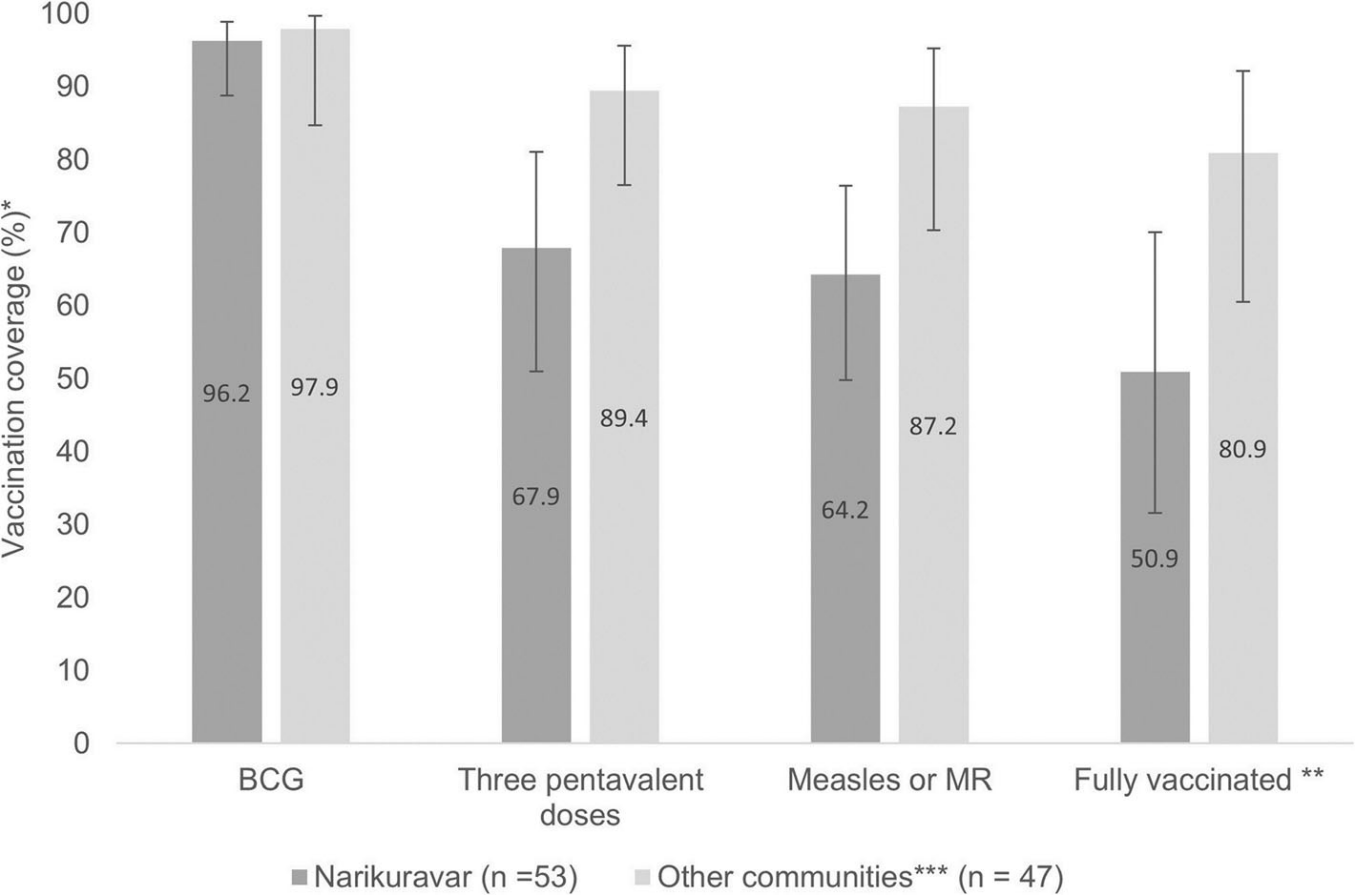
Coverage and vaccination status of children aged 12–23 months by community type in Vellore, southern India (*N* = 100). Presents comparisons in the proportions of children vaccinated with the different antigens between the Narikuravar and non-Narikuravar communities. Legend: * Coverage proportions based on vaccination card or parental recall information. ** Children who received one dose of BCG, three doses each of OPV and pentavalent vaccination and one dose of measles or MR by 12 months of age *** Other communities include the Irulars, brick-kiln and stone quarry worker communities

In the univariate analysis, children who had a vaccination card were more likely to be fully vaccinated compared to those without a vaccination card available during the survey (77% versus 53%, *p* = 0.006, Table 3). Children from non-Narikuravar communities (Irular, brick kiln, and stone quarries) were more likely to be fully vaccinated than children from Narikuravar communities (81% versus 51%, *p* = 0.022). Children from non-Narikuravar communities especially had a higher coverage of pentavalent and measles vaccination compared to children from the Narikuravar communities (Fig. 1). Children with educated mothers (primary schooling or higher versus no formal education) and with mothers who were homemakers (compared to daily wage or salaried employees) or fathers who were daily wage laborers (compared to salaried employees) were also more likely to be fully vaccinated (Table 3). In addition, parents’ familiarity with the recommended immunization schedule for their children, receiving information about the immunization schedule during antenatal visits, and receiving a financial incentive for up-to-date vaccination (with three pentavalent doses) were positively associated with children’s vaccination status (Table 3).

On multivariate analysis, children whose mothers were wage earners (adjusted Prevalence Odds Ratio (aPOR): 0.21, 95% CI = 0.07–0.64), or salaried/small business owners (aPOR: 0.18, 95% CI = 0.04–0.73) were significantly less likely to be fully vaccinated than children who had homemaker mothers (Table 5). The positive association between parental familiarity with the recommended childhood immunization schedule and children’s vaccination status remained in the multivariate analysis but was no longer statistically significant (aPOR: 2.89, 95% CI = 0.90–9.28). In addition, while children from Narikuravar communities had less than half the odds of being fully vaccinated compared to children from the other surveyed communities, this finding was not statistically significant (aPOR: 0.33. 95% CI = 0.06–1.91). Hosmer and Lemeshow’s goodness-of-fit test yielded a *p*-value = 0.226, indicating that the multivariate logistic regression model fit the data well.

**Table 5:** Multivariate analysis of the parental characteristics associated with children’s vaccination status among communities experiencing disadvantage in Vellore, southern India (*N* = 100)

Characteristic	Categories	***N*** (%)	Prevalence Odds Ratio (95% CI)	
			Unadjusted	Adjusted
**Mother’s education**	No formal education	46 (46.0)	**Ref**	**Ref**
	Primary school or higher	54 (54.0)	3.50 (1.15–10.64)**	0.99 (0.20–4.94)
**Mother’s occupation**	Home maker	51 (51.0)	**Ref**	**Ref**
	Wage earner	17 (17.0)	0.27 (0.09–0.82)**	0.21 (0.07–0.64)**
	Salary earner/small business owners	32 (32.0)	0.11 (0.04–0.31)**	0.18 (0.04–0.73)**
**Father’s occupation**	Unemployed/wage earner	62 (62.0)	**Ref**	**Ref**
	Salary earner/small business owners	38 (38.0)	0.29 (0.12–0.68)**	1.30 (0.40–4.22)
**Community type**	Other communities^a^	47 (47.0)	**Ref**	**Ref**
	Narikuravar	53 (53.0)	0.25 (0.08–0.81)**	0.33 (0.06–1.91)
**Vaccination card**	Not available	49 (49.0)	**Ref**	**Ref**
	Yes	51 (51.0)	2.88 (1.38–5.99)**	1.59 (0.61–4.19)
**I am familiar with the recommended immunization schedule for children**	Not agree (N,DA, SDA)^b^	44 (44.0)	**Ref**	**Ref**
	Agree (SA,A)	56 (56.0)	3.31 (1.09–10.02)**	2.89 (0.90–9.28)*
**Received information about the recommended immunization schedule during antenatal visits**	No or not sure	9 (9.0)	**Ref**	**Ref**
	Yes	91 (91.0)	7.89 (1.86–33.28)**	4.55 (0.58–35.38)
**Incentive for receiving three doses of pentavalent vaccine**	No or not sure	51 (51.0)	**Ref**	**Ref**
	Yes	49 (49.0)	3.07 (1.26–7.49)**	1.18 (0.26–5.28)

* *p* < 0.10

** *p* < 0.05

^a^ Other communities include the Irular, brick kiln, and stone quarry worker communities

^b^ *SA* Strongly agree, *A* Agree, *N* Neutral, *DA* Disagree, *SDA* Strongly disagree

### Focus group discussions

Forty-three parents (22 mothers and 21 fathers) participated in the FGDs conducted in Narikuravar, Irular, and stone quarry worker communities. Each focus group had 7–8 parents, and the FGDs lasted between 25 and 40 min with a mean duration of 31 min. All the “5As” domains were discussed organically by parents in the FGDs, and these findings are summarized below.

### Access

Parents did not report issues with accessing routine immunization services in their regular places of residence; however, parents from Narikuravar and Irular communities expressed difficulties getting their children vaccinated when travelling “out of town”. Parents from Irular communities discussed that this is an important reason for missed or delayed vaccination doses for children.

*My child has missed vaccines. We were out of town for a long time while she was younger. As far as I know, she only received two vaccines.*

*(Father, Irular community)*

*There are parents [in the community] who delay vaccines by a month or two, may be because they travel out of the town [ … ].*

*(Mother, Irular community)*

Parents from the Narikuravar settlement provided greater detail on the specific time of the year they were likely to travel out of town.

*After new year we go out (January-March), during Pongal [a southern Indian harvest festival] we stay out for 20 to 25 days.*

*(Father, Narikuravar community)*

### Affordability

Parents from Irular and stone quarry communities discussed the benefit of receiving routine childhood vaccines for free, at times comparing it to vaccines available in private clinics or hospitals, which they felt were for the more affluent.

*The government is giving vaccination free, if we had to get those vaccines in private clinics it would cost us 1000 or 2000 INR [15 – 30 USD], we cannot afford that, so we take the vaccines given by the government.*

(Father, Irular community)

The convenient timing (*a non-financial cost described in the 5As taxonomy*) of routine vaccination sessions was discussed as a facilitator of childhood vaccination uptake, especially by mothers in the Narikuravar community.

*This time [10 – 12 am] is the best for us, if we leave the house by 10, we are able to get the vaccine by 11 and return home.*

(Mother, Narikuravar community)

### Awareness

There was widespread understanding of the general benefits of childhood vaccination, with parents describing the utility of vaccines to prevent diseases and keep their children healthy. A mother described the benefits of vaccination in general and specific terms, referring to the protection her child had received against measles.

*If we vaccinate our children, they are healthy and well, no problems will come to them. Many other children get measles, but my child does not have it because she has been vaccinated.*

(Mother, Narikuravar community)

Fathers from the Irular and quarry worker communities, however, commonly expressed their desire for more information on how diseases occur, how vaccines work, and if there were any other benefits or risks from vaccination that they needed to know.

*We want to know more about the diseases, how they come and how vaccines help reduce them, this advice would be very helpful to us. [ … ] We want to keep our children safe and healthy, that is very important to us.*

(Father, Irular community)

*The problem in villages like ours is that fathers generally go for work 6 days a week, we are free on Sundays only. So we don’t get a chance to go for vaccination sessions. Most people don’t have much awareness about vaccines.*

(Father, Quarry worker community)

Parents generally referred to vaccines (in the routine immunization schedule) in terms of the diseases vaccines protect their children against. A few parents highlighted the role of village health nurses (VHNs) in disseminating vaccination-related information.

*The nurse sometimes seats a few of us parents and explains why the vaccine is being given and when the next vaccine is due. They tell us where the vaccine must be given also [site of administration].*

(Mother, Quarry worker community)

A few parents across the communities highlighted the need for more detailed information on the routine immunization schedule and the need for regular knowledge-sharing sessions at a convenient time for their community.

*Before they [VHN/doctor] vaccinate our children, they do not give us enough information. For example, they say, this is the 1.5-month vaccine, they do not tell us the name or what it is for, similarly with the 2.5- and 3.5-month vaccines [the mother is referring to the pentavalent vaccine].*

(Mother, Quarry worker community)

Parents from the Narikuravar community also discussed their lack of awareness about getting their children vaccinated when travelling out of town for work.

*We do not know anything there, it is a new place. We do not know where to get it done [vaccinations]. We wait till we return back home, and get our child vaccinated then.*

(Mother, Narikuravar community)

### Acceptance

Parents across the communities were largely accepting of vaccinations for their children. Many mothers revealed being the primary decision-makers for vaccinating their children and appeared proactive in following up with vaccinations for both their child and other children in the neighborhood. A Narikuravar mother described the need for vaccinating all children, seemingly assuming the role of a “vaccination advocate” in her community.

*If there is anyone [an unvaccinated child] like that, we tell them to vaccinate their child. It is good for the child. There are 12 months in a year and 24 hours in a day, what if anything happens to the child at that time? It is important to vaccinate children to keep them protected at all times.*

(Mother, Narikuravar community)

Parents from the Narikuravar and Irular communities at times expressed fears due to negative news reports about specific childhood vaccines in the media or from potential side-effects following vaccination as reasons for children not being vaccinated.

*Many parents got scared because of that [the news], some did not want to give their children polio drops. People saw some news on TV and some video, and got afraid, will anything like this happen to our children?*

(Mother, Narikuravar community)

*There are many parents who are afraid that their child may get fever after vaccination, or that their child may have some defects.*

(Mother, Irular community)

### Activation

The importance of prompts and reminders for childhood vaccinations was discussed by a small number of Narikuravar and Irular parents. Telephonic reminders and house visits by the VHNs were discussed as facilitating childhood vaccination uptake.

[ … ] *Even if we miss immunization sessions in the Anganwadi [public childcare centers], she [the VHN] comes in search of the specific houses with such children and organizes special sessions to get them vaccinated the following day.*

(Father, Irular community)

## Discussion

This study found that full immunization coverage among young children from the communities experiencing disadvantage in Vellore was 65% and 77% using information based on vaccination cards or parental recall and vaccination cards alone, respectively. These coverage estimates are similar to recent studies among migrant (67%), tribal (78%), and slum (72%) populations in other parts of India, but lower than the prescribed Mission Indradhanush target of 90% [9, 29–31]. Previous studies from India predominantly report coverage estimates combining vaccination cards and parental recall information [12, 22, 29, 32–35]. We calculated vaccination coverage estimates using information from vaccination cards or parental recall and for vaccination cards alone following EPI guidelines and due to the low proportion of children with vaccination cards available during the survey (~ 50%). Our study found higher vaccination coverage for children with vaccination cards (*n* = 51) than the entire sample (*n* = 100), which was contrary to expectation as combining vaccination cards and parental recall generally provides the highest estimate of vaccination coverage [20]. A large study covering all districts in the state of Tamil Nadu also found lower full vaccination coverage (among children aged 12–23 months) when combining vaccination card and parental recall information than for vaccination cards alone in rural (78.6% versus 80%) and urban (73% versus 73.4%) regions in five districts (including Vellore) [36]. The accuracy of parental recall is often reduced by parents forgetting the number or types of vaccination given to their children, providing socially desirable responses, or receiving incorrect information on the immunization schedule from health workers [37]. Therefore, there is a need to improve vaccination card retention and explore alternate sources of vaccination histories such as provider-maintained records to improve the accuracy of vaccination coverage estimates for children from disadvantaged communities in Vellore.

We observed an important difference in full vaccination coverage between children from the Narikuravar and Irular, brick kiln, and stone quarry worker communities (51% vs. 81%, respectively). The coverage of pentavalent and measles doses was especially lower among Narikuravar children than children in the other communities (Fig. 1), which is concerning considering reports of measles, rubella, and diphtheria outbreaks in other parts of the country [38, 39]. Most Narikuravar parents in our study reported that their children were born in public facilities (75%, *n* = 40) and that they possessed vaccination cards for their children (81%, *n* = 43), indicating sufficient access to public health services. Another study among Narikuravar women in Chennai, Tamil Nadu, revealed that women had no issues accessing vaccination for their children [15]. However, parents from the Narikuravar and Irular communities revealed difficulties in accessing routine immunization services when travelling out of town for work. This finding is similar to a study among Gypsy and Irish Traveller communities in the United Kingdom, where some parents discussed difficulty getting appointments for children’s vaccinations when away from their usual residence [40]. Parents in this UK study also discussed that scheduling childhood vaccinations around travel commitments, receiving reminders about due vaccines through short message service (SMS) text messages or healthcare workers, and having access to walk-in clinics (not requiring prior appointments) helped them catch up on missed vaccinations for their children [40]. While our study was not designed to compare vaccination coverage estimates between the individual communities, these preliminary findings suggest the need for improving awareness on how and where the Narikuravar (and Irular) communities can access routine vaccinations when away from their regular residence and scheduling catch-up appointments for due vaccination doses.

Children whose mothers were wage earners, or salaried/small business owners were significantly less likely to be fully vaccinated than children who had homemaker mothers in the multivariate analysis. This negative association between maternal employment and children’s vaccination status appears counterintuitive as studies among disadvantaged communities and the general population from India and other countries report higher vaccination rates for children with working mothers [35, 41, 42]. Maternal employment is hypothesized to improve uptake by removing financial obstacles to vaccination but may also contribute to missed vaccination appointments due to work commitments [35, 42]. Parents from Roma communities in the United Kingdom reported missing immunization appointments for their children due to long working hours [43]. This may have been true for the children with working mothers (49%, *n* = 49) in our study; mothers from the Narikuravar communities discussed conveniently timed sessions as facilitating childhood vaccination uptake in the focus groups. Having flexible immunization appointments (within 1–2 days of the original appointment) and widespread use of SMS text-based and face-to-face reminders were reported to improve childhood vaccination uptake among communities experiencing disadvantage in the United Kingdom [43]. The district health authorities in Vellore could collaboratively plan immunization sessions based on the availability of parents, and ongoing telephonic or face-to-face reminders by village health nurses (and other health workers) are important to ensure timely childhood vaccinations in these communities.

Just over half (56%) of the parents strongly agreed or agreed that they were familiar with the recommended vaccination schedule for their children in the household survey. We also found a positive but non-significant association between parental familiarity with the vaccination schedule and children’s vaccination status in the multivariate analysis. Many previous studies among migrant, tribal, and slum-dwelling communities in India report a lack of parental awareness about the vaccination schedule, place of vaccination, and the need for vaccination frequently as reasons for children being partially vaccinated or unvaccinated [12, 29, 31, 35, 44]. Parents who participated in the FGDs in our study were generally aware of the benefits of vaccination and could list a few vaccines from the routine immunization schedule. However, many parents were dissatisfied with the depth of vaccination-related information provided by health workers (village health nurses and doctors). Fathers, in particular, requested more information on the benefits and risks of vaccination and the specific vaccines available in the routine immunization schedule for their children. Community-based health education through village meetings or home visits has been shown to improve the coverage of DTP3 vaccination among children in a Cochrane review [45]. While mothers generally receive information on childhood vaccines during antenatal visits [46], periodic community-based health education campaigns could educate better and engage the fathers of disadvantaged communities in Vellore.

Around a fifth (22%) of the parents were hesitant (strongly hesitant, hesitant, or neutral) towards childhood vaccines in the household survey. Although parental vaccine hesitancy was not linked to childhood vaccination uptake in the multivariate analysis, it is an important barrier to children being fully vaccinated in migrant and slum-dwelling communities in India [12, 30, 47]. Fear of vaccine side-effects is a frequently cited reason for children from Roma and Traveller communities in Europe being under-vaccinated [48]. A few parents from the Narikuravar and Irular communities expressed fears due to negative news reports about certain vaccines (one parent mentioned the polio vaccine) and common side-effects following vaccination such as fever or body pain. Parents (in the FGDs) could not remember any details of these news reports but were probably referring to a report about two deaths among children in the Theni and Dindigul districts of Tamil Nadu, wrongly linked to the oral polio vaccine in 2014 [49]. These deaths were due to suffocation and aspiration resulting from children being overfed post-vaccination [49]. Community-based health education campaigns can also build confidence in vaccines by combating the prevalent rumors and misconceptions regarding childhood vaccines and educating parents on managing the common side effects following immunization.

Our study had a few limitations that are important to consider. The findings from the household survey must be interpreted in the light of its non-probabilistic design and small sample size, which limits generalizability to the other disadvantaged communities in southern India. The use of snowball sampling may have resulted in participants being more inter-dependent and missing outlier families, further impacting the accuracy of our survey estimates and the generalizability of the survey findings [23]. We accounted for the clustering of children within the individual communities to provide design-adjusted standard errors (and 95% CIs) for the proportions and estimates presented in this study. The multivariate analysis may also have been underpowered to detect statistically significant associations between the different parental characteristics and children’s vaccination status due to the small sample size. Next, there were fewer brick kiln and stone quarry communities than expected in Vellore, possibly due to changes in government regulations toward quarry workers and bonded labor at brick kilns [50, 51]. As a result, we could not estimate vaccination coverage for each community due to the low number of eligible children. Data saturation could not be achieved in the focus groups due to the limited number of meetings conducted with parents in the different communities. While we are unable to comment on the range of responses that may have been obtained by conducting more meetings, we attempted to triangulate the findings from the focus groups and the household survey using the “5As” taxonomy domains and only use the qualitative findings to elaborate on those from the household survey. Finally, although the important responses from participants were clarified during the focus groups, we did not perform adequate cultural clarifications (during the analysis of the survey and FGD data) from the members of each community due to logistical and time constraints. This may introduce a reporting bias while discussing the possible reasons for community-held perceptions, attitudes, or behavior towards childhood vaccinations.

The limitations notwithstanding, our survey provides the most recent estimate of routine vaccination coverage for children from the Narikuravar, Irular, and migrant communities in Vellore. Despite the individual limitations of our household survey and focus group discussions, using a mixed-methods approach helped identify and describe the important parental characteristics linked to childhood vaccination uptake among disadvantaged communities in Vellore. The survey data were collected using the KoBo Toolbox, an open-source application for Android™ devices, which helped decrease the possibility of data-entry errors with pre-programmed range checks and skip patterns for the electronic questionnaire. Furthermore, using the “5As” taxonomy to outline study questions and map responses from the FGDs helped identify important barriers and facilitators of routine childhood vaccination, informing targeted and contextual interventions to improve vaccination uptake in these communities.

## Conclusions

Recent estimates of routine immunization coverage among young children from communities experiencing disadvantage in India are lacking. We found lower full vaccination coverage (65–77%) among children aged 12–23 months in Vellore than the prescribed Mission Indradhanush target of 90%. Children whose mothers were wage earners, or salaried/small business earners were less likely to be fully vaccinated than children with homemakers mothers in the household survey. In the focus groups, parents identified difficulties in accessing routine immunization when travelling for work (reported by the Narikuravar and Irular communities), showed important knowledge gaps regarding the benefits and risks of vaccination, and fears due to negative media reports and common-side effects following vaccination. While larger studies are needed to validate our findings, our study findings suggest the need for targeted and contextual interventions to improve routine immunization uptake among children from the communities experiencing disadvantage in Vellore.

## Supplementary Information

**Additional file 1.**

## Data Availability

The data that support the findings of this study are available from the Christian Medical College, Vellore, Tamil Nadu, India, but restrictions apply to the availability of these data due to the sensitive nature of the study topic and populations interviewed, and thus are not publicly available. Data may be made available by the corresponding author upon reasonable request.

## Abbreviations

UIP: Universal Immunization Program
MI: Mission Indradhanush
NFHS: National Family Health Survey
IRB: Institutional Review Board
BCG: Bacillus Calmette-Guerin
OPV: Oral Polio Vaccine
MR: Measles-Rubella
SD: Standard Deviation
FGD: Focus Group Discussion
VHN: Village Health Nurse
