# Supplementary material for: Vaccination coverage and the factors influencing routine childhood vaccination uptake among communities experiencing disadvantage in Vellore, southern India: a mixed-methods study
Source: BMC Public Health. 2021 Oct 7;21:1807. doi: 10.1186/s12889-021-11881-8 (PMC8499461; doi:10.1186/s12889-021-11881-8)
Supplement: Supplementary file 1 — Additional file 1. [file 12889_2021_11881_MOESM1_ESM.docx]

**SUPPLEMENTAL MATERIAL**

**Supplementary Table 1:** STROBE Checklist

|  | Item No | Recommendation | Page No |
| --- | --- | --- | --- |
| **Title and abstract** | 1 | (*a*) Indicate the study’s design with a commonly used term in the title or the abstract | 1 |
|  |  | (*b*) Provide in the abstract an informative and balanced summary of what was done and what was found | 2-3 |
| Introduction | | | |
| Background/rationale | 2 | Explain the scientific background and rationale for the investigation being reported | 4-5 |
| Objectives | 3 | State specific objectives, including any prespecified hypotheses | 5 |
| Methods | | | |
| Study design | 4 | Present key elements of study design early in the paper | 5-7 |
| Setting | 5 | Describe the setting, locations, and relevant dates, including periods of recruitment, exposure, follow-up, and data collection | 5-7 |
| Participants | 6 | (*a*) Give the eligibility criteria, and the sources and methods of selection of participants | 6-7 |
| Variables | 7 | Clearly define all outcomes, exposures, predictors, potential confounders, and effect modifiers. Give diagnostic criteria, if applicable | 8-10 |
| Data sources/ measurement | 8 | For each variable of interest, give sources of data and details of methods of assessment (measurement). Describe comparability of assessment methods if there is more than one group | 8 |
| Bias | 9 | Describe any efforts to address potential sources of bias | 8-10 |
| Study size | 10 | Explain how the study size was arrived at | 7 |
| Quantitative variables | 11 | Explain how quantitative variables were handled in the analyses. If applicable, describe which groupings were chosen and why | 9-10 |
| Statistical methods | 12 | (*a*) Describe all statistical methods, including those used to control for confounding | 9-10 |
|  |  | (*b*) Describe any methods used to examine subgroups and interactions | Not applicable |
|  |  | (*c*) Explain how missing data were addressed | Not applicable |
|  |  | (*d*) If applicable, describe analytical methods taking account of sampling strategy | Not applicable |
|  |  | (*e*) Describe any sensitivity analyses | Not applicable |
| Results | | | |
| Participants | 13 | (a) Report numbers of individuals at each stage of study—eg numbers potentially eligible, examined for eligibility, confirmed eligible, included in the study, completing follow-up, and analysed | Not applicable |
|  |  | (b) Give reasons for non-participation at each stage | Not applicable |
|  |  | (c) Consider use of a flow diagram | Not applicable |
| Descriptive data | 14 | (a) Give characteristics of study participants (eg. demographic, clinical, social) and information on exposures and potential confounders | 13-14, Table 3 |
|  |  | (b) Indicate number of participants with missing data for each variable of interest | Not applicable |
| Outcome data | 15 | Report numbers of outcome events or summary measures | 13-14, Table 3 & Table 4 |
| Main results | 16 | (*a*) Give unadjusted estimates and, if applicable, confounder-adjusted estimates and their precision (eg, 95% confidence interval). Make clear which confounders were adjusted for and why they were included | 15, Table 3 & Table 5 |
|  |  | (*b*) Report category boundaries when continuous variables were categorized | Not applicable |
|  |  | (*c*) If relevant, consider translating estimates of relative risk into absolute risk for a meaningful time period | Not applicable |
| Other analyses | 17 | Report other analyses done—eg analyses of subgroups and interactions, and sensitivity analyses | Table 5, Figure 1 |
| Discussion | | | |
| Key results | 18 | Summarise key results with reference to study objectives | 22-26, 28 |
| Limitations | 19 | Discuss limitations of the study, taking into account sources of potential bias or imprecision. Discuss both direction and magnitude of any potential bias | 26-27 |
| Interpretation | 20 | Give a cautious overall interpretation of results considering objectives, limitations, multiplicity of analyses, results from similar studies, and other relevant evidence | 22-28 |
| Generalisability | 21 | Discuss the generalisability (external validity) of the study results | 26-27 |
| Other information | | | |
| Funding | 22 | Give the source of funding and the role of the funders for the present study and, if applicable, for the original study on which the present article is based | 30 |

**Cross-sectional survey questionnaire (paper-version)**

**Thematic guide for the focus group discussions**

1. What are your **views on vaccination** for your child in general? (*Probes:* *Are vaccines necessary or can they be done without?)*
2. What do you see as the **benefits of vaccinating your child**? (*Are there any risks with government vaccination? What are your suggestions to reduce your concerns about vaccinations?*)
3. Who helps you with **making decisions for vaccination** for your child (*Who do you trust the most and why?*)
4. How is your **relationship with the healthcare provider** (Doctor/Nurse/ASHA/ANM) who generally provides vaccination for your children? (*Are you comfortable to clarify any issues you may have?*)
5. Are you satisfied with the **information on the benefits and risks** of particular government vaccines that your healthcare provider (Doctor/Nurse/ASHA/ANM) provides you before vaccination? (*Is there any way this information can be improved?*)
6. Are you aware of **any children from specific communities or households** who do not take all government vaccines or refuse certain vaccines in your area? (*What are some of the reasons why?*)
7. Are you satisfied with the **childhood vaccination services the government provides**? (*What are the benefits you have received? Are there any aspects that you would like improved?*)
